# Significance of metastamiR-10b in breast cancer therapeutics

**DOI:** 10.1186/s43046-022-00120-9

**Published:** 2022-05-02

**Authors:** Apexa Raval, Jigna Joshi, Franky Shah

**Affiliations:** Molecular and Diagnostic Research Lab-4, Department of Cancer Biology, The Gujarat Cancer & Research Institute, Ahmedabad, India

## Abstract

**Background:**

Breast cancer is a fatal disease and a major reason of cancer associated death in females. Many factors along with miRNA are responsible for the development and the progression of the disease. The miRNA plays a very crucial role in the regulation of the genes. MicroRNAs are of three major types—oncomiRs, tumor suppressive miRNAs, and metastamiRs.

**Main body:**

MicoRNA-10b is a prometastatic microRNA targeting various genes that facilitates multiple outcomes such as metastasis, increased capacity for invasion, proliferation and migration, increased epithelial-mesenchymal transformation, angiogenesis, and therefore exhibits worse clinical outcomes. It is found to be upregulated in various malignancies and is thus to be considered as the possible therapeutic candidate.

**Conclusion:**

The therapeutic delivery of miR-10b antagonists (antagomiRs) and/or knockdown of miRNA is beneficial in reducing tumor growth. Additionally, combination therapy which includes antisense oligonucleotides using miR-10b can function as an effective approach to tumor regression and drug resistance reversal.

**Graphical abstract:**

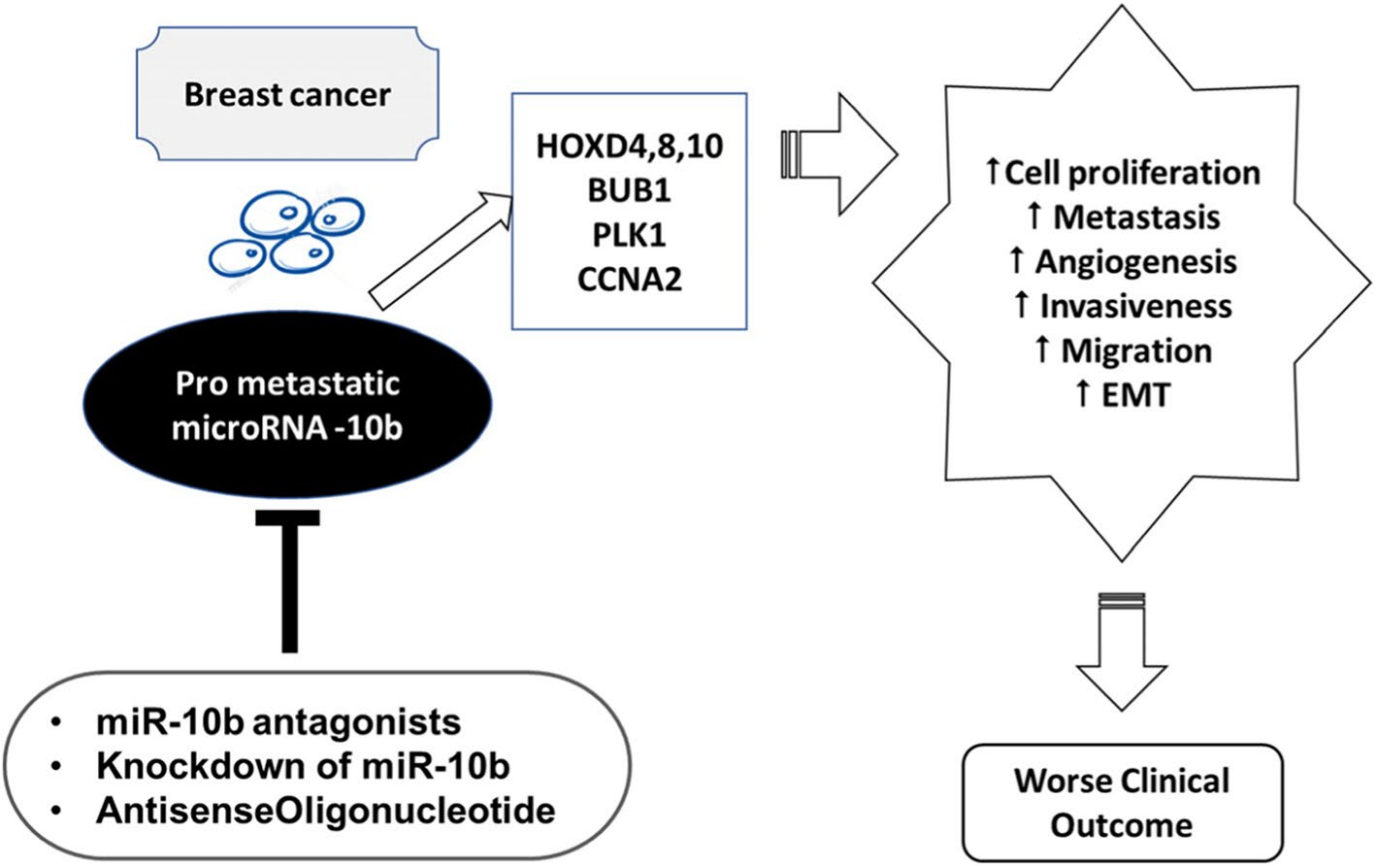

## Background

Breast cancer is a complex and heterogeneous disease and is highly illusory in its progression mechanism. It is the second leading cause of death amongst women worldwide. According to Globocan 2018 incidence of breast cancer is 11.6% of all new cases and mortality is 6.6% of all cancer death (https://www.uicc.org/new-global-cancer-data-globocan-2018).

Different factors including growth factors, cytokines and chemokines, pro-angiogenic factors, extracellular matrix-remodelling molecules, multiple epithelial–mesenchymal transition (EMT)-inducing transcription factors, as well as certain miRNAs are involved in the development and progression of the disease [1]. Hence, there is an utmost need to recognize the molecular signature which provides definite support in the field of breast cancer diagnosis and therapy. The miRs are one of the key molecular regulators that play an important role in the pathogenesis of the breast cancer and metastasis [2].

### Role of miRNAs in breast cancer: oncogenic miRs, tumor suppressor miRs, and metastamiR

The role of miRNA dysregulation was first demonstrated in 2005, following that many studies have since found several different miRNAs which are being deregulated in breast cancer [3]. Among all known miRNAs, some act as oncogenic miRNAs by suppressing tumor suppressor genes while some of miRs exhibit tumor suppressor properties by downregulating oncogenic genes [4].

An overexpression of an oncogenic miRNA would lead to tumor formation and progression by eliminating the expression of a miRNA-target tumor suppressor gene [4].

#### Tumor-suppressive micro RNAs

These miRNAs target mRNAs of various oncogenes and downregulate their levels. Any dysregulation of these micro RNAs trigger the formation of the tumor. Most common deregulated tumor suppressive miRNAs in breast cancer are listed in Table 1. Amongst these, Lethal-7 (let-7) family of miRNAs was first identified due to its abundant presence [4, 5] (Table 1). There are certain oncogenic and metastamiRs along with their known targets that promote cell proliferation, angiogenesis, and metastasis.

**Table 1:** List of tumor suppressor, oncogenic, and metastatic miRNAs

Tumor suppressor miRNA [5]		
miRNAs	Targets	Functions
Let-7 family	RAS, HMGA2	Inhibit cell proliferation and mammosphere formation
miR-125	HuR, HER2, ETS1, Cyclin J, MEGF9	Inhibit cell proliferation and invasion
miR-205	ZEB1 and ZEB2	Reduces EMT and metastasis
miR-200 family	ZEB1/2	Reduces EMT and metastasis
miR-206	Cyclin D2	Inhibits Cyclin D2 in MCF-7 cells
miR-34a	Bcl2, SIRT1	Inhibits migration, invasion, and metastasis
miR-31	RhoA, ITGA5, RDX	Reduces invasion and metastasis
**Oncogenic miRNA** [5]		
miR-10b	HOXD10 [5–7]	Promotes cell proliferation, metastasis, and angiogenesis
miR-126	IGFBP2, MERTK, PITPNC1 [6]	Promotes angiogenesis
miR-155	SOCS1, TP53INP1, FOXO3, RhoA [5]	Promotes cell proliferation
miR-21	PTEN, TPM1, PDCD4, Maspin [5, 8]	Promotes cell proliferation
miR-375	RASD1 [9]	Epigenetic modification of tumor suppressor genes
**MetastamiR** [6]		
miR-373	CD44 [10]	Induce metastasis
miR-221/222	TRPS1 [11]	Induce metastasis
miR-520c	CD44 [5, 6]	Induce metastasis
miR-9	SOCS5, E-cadherin [12, 13]	Induce metastasis
miR-632	DNAJB6 [14]	Induce metastasis
miR-105	ZO-1 [15]	Destroys the integrity of tight junctions
miR-223	Mef2c-b-catenin pathway [16]	Enhancing the invasiveness
miR-125b	STARD13 [6]	Promote metastasis
miR-199	ALDH1, FoxP2 [6]	Increase CSC-related traits
miR-494	TGFβ, PTEN [6]	Breast cancer metastasis
miR-182	MIM [6]	Enhances breast cancer cell mobility and invasion
miR-24	PTPN9, PTPRF, pEGFR, ADAM15 [6]	Cell invasion, migration, and metastasis
miR-181a	TGFβ, Bim [6]	EMT, invasion, and migration
miR-148a	Wnt1 and NRP1 [6]	Promote EMT and metastasis

#### Oncogenic and/or metastamiR miRNAs

The micro RNAs that affect the oncogenesis are classified as oncogenic miRNAs. Several such micro RNAs are known in breast cancer too, showing their oncogenic potential by inducing cell proliferation and tumorigenesis and/or metastasis [5] (Table 1). These oncogenic micro RNAs exhibit their function through targeting the genes which are crucial in the said processes (Table 1).

Cancer metastasis is still a fatal challenge. In the last 10 years, significant progress has been made in understanding the functions of specific miRNAs in metastasis. Some of these metastamiRs (Table 1) represent attractive therapeutic targets for cancer treatment. Thus far, there are no effective therapies for treating metastatic cancer [4, 17]. There are several hindering factors that affect the metastatic trials on patients with early-stage cancer. Further the miRNA involvement have been noted in various mechanisms such as regulation of oncogenes, tumor suppressor genes and metastasis genes, cancer stem cell properties, epithelial-mesenchymal transition (EMT), microenvironment, and exosome secretion [18]. Moreover, a single miRNA (or miRNA cluster/family) may play a dual role in the invasion-metastasis cascade.

Thus, there are several metastamiRs and oncomiRs that aid in the development of the metastasis [1]. Among all such miRs, miR-10b has been studied to the greatest degree in breast cancer relative to other types of cancer that enhance the metastatic properties; and its association with the known biomarkers of the breast cancer is already extensively studied [19].

Hence, the detailed discussion about miR-10b is carried out here.

#### miR-10b

Among the deregulated microRNAs, miR-10b is considered as the pro-metastatic microRNA in breast cancer [7]. miR-10b is located on the chromosome-2 with the cluster of HOXD gene in intergenic region between HOXD4 and HOXD8. miR-10b was first identified by Iorio et al. [20] as one of the most significantly downregulated microRNA in primary breast tumors compared to the normal breast samples. While in another study, miR-10b was found to act as a tumor suppressor miR. Subsequently, Weinberg’s group reported a contradictory concept that miR-10b could act as a metastasis-associated miRNA (*metastamiR*) in advanced breast tumors [21, 22].

miR-10b targets various genes such as HOXD4, BUB1, PLK1, and CCNA2. The loss of HOXD10 expression was observed in breast tumors that lead to malignancy. Restored expression of HOXD10 in MDA-MB-231 cells has been found to impair migration and invasion in vitro as well as tumor progression in vivo [7]. Another target gene of miR-10b is RhoC, that was reported to promote tumor metastasis in distinct carcinomas by stimulating the activity of series of kinases including AKT, and mitogen-activated protein kinase (MAPK) [22]. miR-10b is directly correlated with the progression of glioblastoma and breast cancer. Increased expression of miR-10b is associated with multiple outcomes such as metastasis, increased invasive potential in vitro and in-vivo, migration, increased epithelial-mesenchymal transition, angiogenesis, and increased proliferation. These changes result in worse clinical outcomes, including increased tumor size, advanced clinical stage, and short relapse-free survival [19].

Recently Meerson et al. [23] discovered that miR-10b may be a mediator between obesity and cancer in post-menopausal women, regulating several known cancer-relevant genes. Moreover, miR-10b expression may have diagnostic, prognostic [24], and therapeutic implications for the incidence and prognosis of breast cancer in obese women. The circulating miR-10b can be used as a biomarker in breast cancer [24, 25]. Correlating the miR-10b expression with the clinical characteristics in patients with different molecular subtypes. The positive expression rate of miR-10b was lowest in patients with Luminal B breast invasive ductal carcinoma [26].

miR-10b shows positive correlation with HER2 status and negative correlation with ER, PR status, and this association is the predictor of the tumor aggressiveness. Specifically, it was demonstrated that the stable overexpression of miR-10b in MCF-7 cells resulted in higher self-renewal and expression of genes that promote stemness property and epithelial-mesenchymal transition [19].

Ma et al. [12] in his study showed that Twist directly binds upstream of the miR-10b gene and regulates its expression. Thus, miR-10b is induced by Twist and mediates its effects by targeting HOXD10 that plays a role in tumor invasion and metastasis [7].

The study of the Asian population wherein meta-analysis was carried out considering 9 different studies reveal that in Asian population upregulation of miR-10b was significantly correlated with the metastasis status and suggest the potential use of miR-10b as a molecular marker as well as for the assessment of the prognosis of the cancer patients [27].

On the other hand, a study on the West Sumatran women indicates that miR-10b expressions were lower in breast cancer as compared to the fibroadenoma tissues. The study was conducted from the primary tumors, and it was suggested that the results can be used as a guide for oncologists later to investigate the development of metastasis [28].

The study of the Lebanese women demonstrated the notable downregulation of miR-10b. Further miR-10b was significantly underexpressed in ER/PR-negative tumors compared to ER/PR-positive tumors [29].

The study on the Egyptian breast cancer patients suggest that miR-10b was upregulated in tumor tissue compared to adjacent normal tissue (ANT) and the overexpression of miR-10b increased the level of MMP-2 and MMP-9 genes. Therefore miR-10b can be used in breast cancer prognosis [30]. 

Thus, miR-10b is a metastamiR and may be considered as the potential therapeutic target.

Several types of in vivo miRNA antagonists are being developed as antagomirs. The miR-10b antagomir had a potent and highly specific metastasis-suppressing effect in the mouse model. Moreover, it also blocks the dissemination of cancer cells from the primary tumor and prevents the toxicity to the normal tissue [25]. The main promise for developing an agent such as antagomir-10b as a potential therapy would be whether it can be added during treatment starting in the early stages as a prophylactic therapy against future metastasis formation. Since antagomir-10b does not shrink a primary tumor, it should be combined with other anti-tumor drugs.

With respect to its role in immune cell regulation, miR-10b inhibition showed greater NKG2D-mediated killing of tumor cells in vitro and greater clearance of tumor in vivo [31]. From these examples, it becomes clear that as therapeutic target, miR-10b has a diverse set of possible effects which all lend to improved clinical outcomes. This is a promising route of treatment. Locked nucleic acid (LNA) antagomirs were delivered to metastatic cells by dextran-coated iron oxide nanoparticles (termed MN-anti-miR10b). These MN-anti-miR-10b prevented the genesis of new metastases, following intravenous injection [32]. Evidence in favor of the choice of miR-10b as a therapeutic target comes from a pivotal study, which explored the effects of complete knockout of miR-10b in murine models of breast cancer [33]. As it is essential to minimize off-target effects, miR-10b was shown to be dispensable for normal development but essential for tumorigenesis in miR-10b-deficient mice. These mice showed delayed tumorigenesis and suppressed EMT, intravasation, and metastasis [33]. This demonstrates the value of miR-10b as a therapeutic target with potentially minimal off-target effects.

## Conclusions

The cancer-related mortality (~ 90%) is caused by metastasis which can be rarely prevented using current cancer treatment approaches. MicroRNA-10b is a metastamiR found to be involved in the processes leading to the metastasis and hence best studied in breast cancer. Upregulation of miR-10b exhibits worse clinical outcomes and hence is considered as the potential therapeutic target. Application of miR-10b antagonists or knockdown of this miRNA are the promising agents that reduce the tumor growth and cell proliferation. Hence, these targeting approaches may be beneficial for many incurable cancers. Moreover, the early usage of these approaches with conventional therapy may impede the development of metastasis and enhance the survival of breast cancer patients.

## Data Availability

Not applicable.

## Abbreviations

EMT: Epithelial–mesenchymal transition
let-7: Lethal-7
MAPK: Mitogen-activated protein kinase
LNA: Locked nucleic acid
